# Sustainable Development of Chitosan/*Calotropis procera*-Based Hydrogels to Stimulate Formation of Granulation Tissue and Angiogenesis in Wound Healing Applications

**DOI:** 10.3390/molecules26113284

**Published:** 2021-05-29

**Authors:** Muhammad Zahid, Maria Lodhi, Zulfiqar Ahmad Rehan, Hamna Tayyab, Talha Javed, Rubab Shabbir, Ahmed Mukhtar, Ayman EL Sabagh, Robert Adamski, Mohamed I. Sakran, Dorota Siuta

**Affiliations:** 1 Department of Chemistry, University of Agriculture, Faisalabad 38000, Pakistan; rmzahid@uaf.edu.pk (M.Z.); mk0441021@gmail.com (M.L.); hamna7575@yahoo.com (H.T.); 2 Department of Materials, National Textile University, Faisalabad 37610, Pakistan; 3 College of Agriculture, Fujian Agriculture and Forestry University, Fuzhou 350002, China; mtahaj@fafu.edu.cn (T.J.); rubabshabbir28@gmail.com (R.S.); 4 Department of Agronomy, University of Agriculture, Faisalabad 38040, Pakistan; ah.mukhtar85@gmail.com; 5 Department of Agronomy, Faculty of Agriculture, Kafrelsheikh University, Kafrelsheikh 33156, Egypt; ayman.elsabagh@agr.kfs.edu.eg; 6 Department of Field Crops, Faculty of Agriculture, Siirt University, Siirt 56100, Turkey; 7 Faculty of Process and Environmental Engineering, Lodz University of Technology, 90-924 Lodz, Poland; robert.adamski@p.lodz.pl; 8 Biochemistry Section, Chemistry Department, Faculty of Science, Tanta University, Tanta 31527, Egypt; msakran@ut.edu.sa; 9 Biochemistry Department, Faculty of Science, University of Tabuk, Tabuk 47512, Saudi Arabia

**Keywords:** hydrogels, chitosan, granulation, wound healing, sustainability, *Calotropis procera*

## Abstract

The formation of new scaffolds to enhance healing magnitude is necessarily required in biomedical applications. Granulation tissue formation is a crucial stage of wound healing in which granulation tissue grows on the surface of a wound by the formation of connective tissue and blood vessels. In the present study, porous hydrogels were synthesized using chitosan incorporating latex of the *Calotropis procera* plant by using a freeze–thaw cycle to stimulate the formation of granulation tissue and angiogenesis in wound healing applications. Structural analysis through Fourier transform infrared (FTIR) spectroscopy confirmed the interaction between chitosan and *Calotropis procera*. Latex extract containing hydrogel showed slightly higher absorption than the control during water absorption analysis. Thermogravimetric analysis showed high thermal stability of the 60:40 combination of chitosan (CS) and *Calotropis procera* as compared to all other treatments and controls. A fabricated scaffold application on a chick chorioallantoic membrane (CAM) showed that all hydrogels containing latex extract resulted in a significant formation of blood vessels and regeneration of cells. Overall, the formation of connective tissues and blood capillaries and healing magnitude decreased in ascending order of concentration of extract.

## 1. Introduction

Wound healing is considered to be a promising subject that aims to repair or enrich the functioning of an impaired organ or tissue. As per the World Health Organization (WHO), about 2.8 million people suffered from wounds, and 146,000 deaths occurred due to road traffic accidents and war in Pakistan during 2019 [1]. For this reason, wound bandages are utilized to treat tissue damage and skin injuries that occur due to accidents, ailments, and surgery [2]. Wound healing comprises combined biochemical and cellular actions directed to recovering the potency of damaged muscles with the restoration of mechanical and efficient reliability [3]. It involves the redevelopment of operative skin illustrated by cooperative and vigorous actions including the formation of an extracellular matrix (ECM) following the cell relocation, proliferation, and segregation [4]. The approach of wound healing is to exchange lost or degraded organs or tissues with composite or polymeric scaffolds consisting of the specified characteristics of living cells, with the purpose of tissue redevelopment and return of usual functions [5]. Wound healing agents such as scaffolds and bandages have gained wide importance as they impart primary biological reinforcement to the substituted tissues until sufficient ECM formation occurs [6]. For appropriate wound recovery, injured muscles must be replaced with biocompatible scaffolds. Therefore, in recent years, improvements have taken place in the formulation of new kinds of dressing substances particularly using biocompatible molecules [7,8]. Dressing substances must ensure germ-free and harmless environments to enhance epidermal cell movement and have such flexible and soft properties that make their removal easy after healing [9,10,11]. Advanced dressings consist of foams and films based on polymers and make connections with the surroundings, prevent penetration of microbes, and stimulate epithelization without scar formation [12].

Materials must have the capacity to absorb water over time for adequate wound recovery [13]. With the introduction of moist wound healing theory, hydrogel dressings have gained wide importance because of their potential to maintain and ensure a moist and soft environment [8]. The mechanical properties of hydrogels resemble soft tissues, supporting and enhancing the healing process and copying the morphological and operative features of tissues themselves [14]. Hydrogels are insoluble with 3D structures formed by different hydrophilic polymers such as chitosan, alginate, and cellulose [15]. These consist of crosslinked and semisolid macromolecular networks that are able to absorb significant amounts of biological fluids and moisture contents. Hydrogels based on natural materials are advantageous because of their safe nature, biocompatibility, and biodegradability. Hydrogels are appropriate scaffolds for the healing of all four phases of the wound [16,17].

Being a natural polymer, chitosan is usually employed in wound healing applications [18]. Chitosan is the most important material that boosts granulation, inflammatory cell function, and cell proliferation in wound healing. It has a structural resemblance to glycosaminoglycan, a protein present in the ECM [19], and has the ability to promote histoarchitectural muscle networks. Owing to its macrophage behavior, powerful bactericidal effect, hemostatic properties, biocompatibility, wound healing properties, excellent biodegradability, low toxicity, low manufacturing rate, and high accessibility, chitosan has been widely utilized in the biomedical domain [20,21,22]. Certain groups such as NH_2_ and OH present in the chitosan provide the opportunity of interaction with biological molecules and other polymers [23].

Bioactive dressings grouped with natural or synthetic pharmaceutically active mediators are assumed to enhance wound healing with an artistic exterior. The current emphasis on the preference of bio-based herbal agents in respect to their easy access, negligible toxicity, and multifunctional roles has enabled their incorporation in wound dressings. *Calotropis procera* (sodom apple) was assessed for its wound healing capability depending upon its conventional utility. *Calotropis procera* latex is described as having antimicrobial, wound healing, anti-inflammatory, and analgesic properties [24]. The granulation tissue consists of protein and DNA components that specify protein level formation, and the mutagenic profile and above-stated characteristics of this plant are specifically responsible for wound healing. By significantly expanding epithelization, collagen, synthesis of protein, and DNA, thus causing a decline in cut size, latex of sodom apple meaningfully increased healing progression [25]. Both above-mentioned materials have wound healing and antimicrobial properties. Chitosan has been utilized as a hydrogel and *Calotropis procera* extract has been utilized directly on wound sites [26] to observe the healing process. Both have been utilized separately as bioactive materials. Previously, no hydrogel incorporating *Calotropis procera* latex extract with any combination of polymers has been reported. Therefore, in the current study, we aim to achieve sustainable development of hydrogel incorporating latex extract of *Calotropis procera* in chitosan using freeze-thawing and investigate their chemical interaction, surface morphology, swelling capability, thermal stability, and healing potential.

## 2. Results

### 2.1. Preparation of Hydrogels

**Figure 1:**
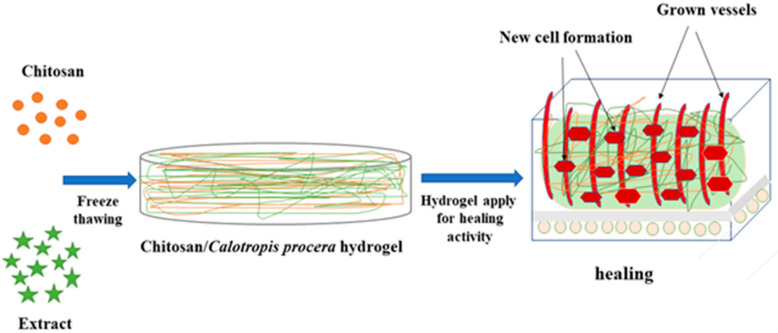
Schematic representation of CS/*Cp*-LE hydrogels.

In this experiment, CS/*Cp*-LE-based hydrogels were synthesized by freeze-thawing to examine their collective impact on wound healing. A schematic illustration of CS/*Cp*-LE hydrogels is shown in Figure 1.

### 2.2. Fourier Transform Infrared Spectroscopy (FTIR)

**Figure 2:**
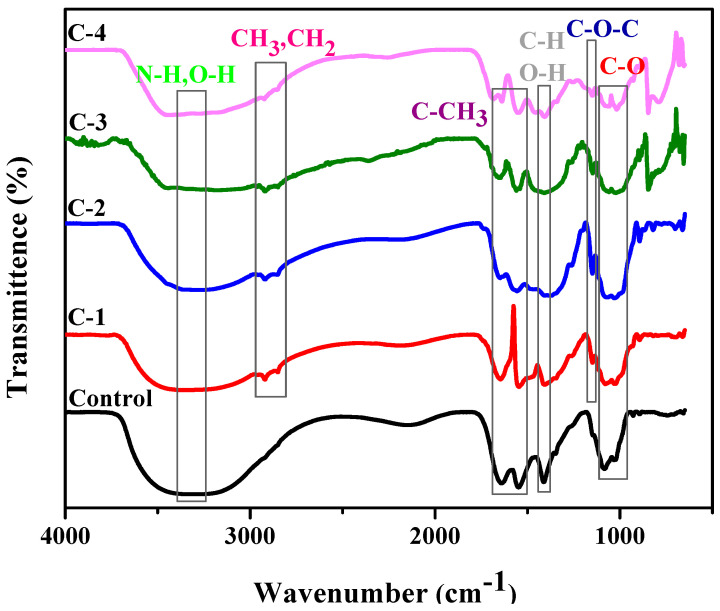
FTIR analysis of CS/*Cp*-LE-based hydrogels.

The chemical interaction between the polymeric material and plant extract was studied by FTIR. Samples C-1, C-2, C-3, and C-4 consisted of different weight percent ratios of 1% concentration solution of CS and *Cp*-LE, namely 60/40, 70/30, 80/20, and 90/10, respectively (Table 1). The control hydrogel showed characteristic peaks of CS (Figure 2). The broad peak at 3300–3350 cm^−1^ was due to N-H and O-H stretching, the peak at 1640 cm^−1^ was assigned to amide I, while the peak at 1553 cm^−1^ showed the presence of C-CH_3_. C-H and O-H deformation peaks were present around 1400 cm^−1^. The band at 1023 cm^−1^ indicated bending of C-O [27]. The band position from 1020–1078 cm^−1^ represents C-O stretching. From the literature, characteristic peaks of *Cp*-LE reviewed show a broad band of 3350 to 3450 cm^−1^ due to the O-H stretching of aliphatic fractions present in polysaccharides [28]. The small peaks at 2919 to 2850 cm^−1^ were assigned to the C-H stretching of CH_3_ and -CH_2_ groups and cellulose fiber contents in *Cp*-LE [29,30]. The peak at 1151 cm^−1^ was due to the C-O bond stretching of cellulose and hemicellulose [31,32,33].

All hydrogels with *Cp*-LE displayed peaks almost at the same positions where peaks of the control sample (Figure 2) and *Cp*-LE were present. The peak in the region of 3150–3350 cm^−1^ represents N-H and O-H stretching as compared to the control sample, and a shift occurs due to the interaction of both CS and *Cp*-LE. The intensity of a N-H stretching peak depends on H-bonding and moisture content. The peak in the range of 2919 to 2850 cm^−1^ in CS/*Cp*-LE hydrogels was due to the C-H stretching of methyl and methylene groups and cellulose fiber contents that confirmed the presence of *Cp*-LE in CS/*Cp*-LE hydrogels. A peak within a range of 1650–1660 cm^−1^ represented amide I, and a peak of 1546 cm^−1^ showed the presence of amide II; minor shifts occurred in their positions due to crosslinking. The deformation peaks of O-H and C-H are present in the 1416 cm^−1^ region in Figure 2. The peak present in the C-1, C-2, C-3, and C-4 hydrogels at 1151 cm^−1^ position was due to the C-O bond stretching of cellulose and hemicellulose that was absent in the control sample. However, FTIR spectra of CS/*Cp*-LE hydrogels indicated a strong interaction between chitosan and *Calotropis procera* latex extract.

### 2.3. Scanning Electron Microscopy (SEM)

**Figure 3:**
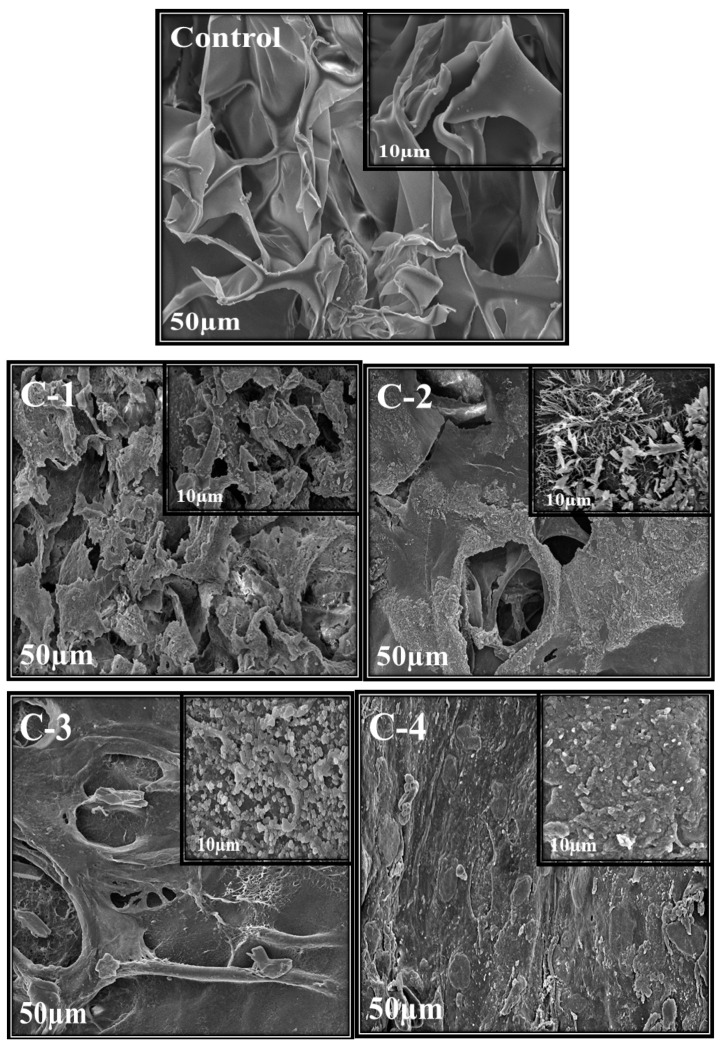
SEM images of CS/*Cp*-LE hydrogels: control, C-1, C-2, C-3, and C-4; at ×1000 and ×2500 magnifications.

Scanning electron microscopy was used to analyze the morphology, structure, and size of the synthesized material. Morphology and porosity of control and all CS/*Cp*-LE hydrogels were examined, and their SEM images are given in Figure 3. The control hydrogel containing only CS polymer showed open pores. These microstructural open pores were interlinked in the whole surface. Control hydrogels contain a highly porous structure and smooth surface consisting of CS material only, as stated in the literature where with the increase in CS ratio, the hydrogel structure becomes looser [34]. SEM images of C-1, C-2, C-3, and C-4 hydrogels with varying concentrations of CS/*Cp*-LE, namely 60/40, 70/30, 80/20, and 90/10, respectively, are shown in Figure 3. All hydrogels containing *Cp*-LE show a less porous surface compared to that of the control hydrogel because their surface is covered with *Cp*-LE content. However, the number of pores in C-1 is greater than that in C-2, C-3, and C-4, possibly due to the decreasing *Cp*-LE ratio of C-2, C-3, and C-4. This was evident from the SEM images: as the *Cp*-LE ratio decreased in the hydrogels, their porosity also decreased in descending order of *Cp*-LE weight percent ratio.

### 2.4. Swelling Analysis

**Figure 4:**
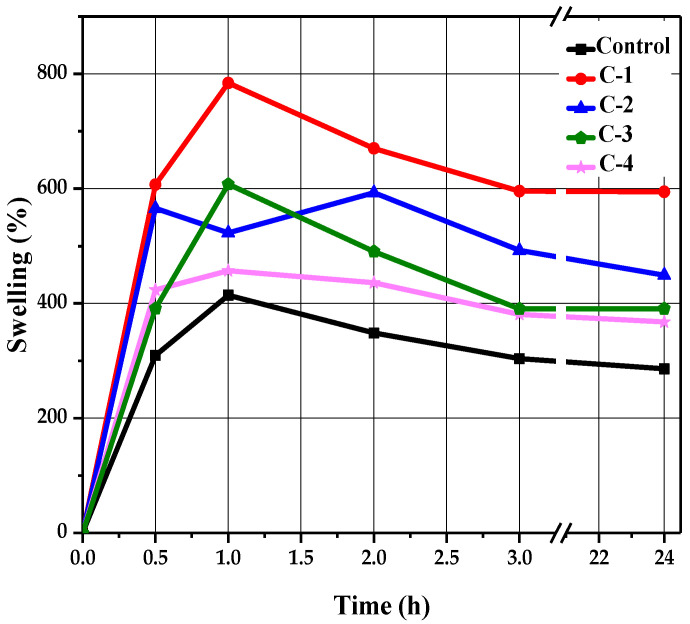
The swelling trend of CS/*Cp*-LE-based hydrogels.

A swelling test was performed to determine the liquid absorption capacity of the hydrogels. Each sample of hydrogel was soaked in a saline solution of NaCl in Petri dishes for 24 h. Swelling behavior was determined at different time intervals of 0.5, 1, 2, 3, and 24 h. After passing each specified time, their swelling percentage values were noted by removing each sample with the help of forceps from the solution and measuring its wet weight using an electronic balance. All samples revealed a time-dependent swelling profile, where their highest absorption value was reached within 1 to 2 h, as shown in Figure 4. It can be observed in Figure 4 that the control hydrogels exhibited minimum water absorption as compared to the hydrogels containing *Cp*-LE, which showed slightly higher liquid absorption. This may be due to the addition of *Cp*-LE in the hydrogels.

The control sample reached a maximum swelling value of 414% within 1 h (Figure 4). After that, no significant changes occurred, but low absorption values were apparent compared to hydrogels with *Cp*-LE. As the *Cp*-LE quantity of C-1, C-2, C-3, and C-4 hydrogels increased, so did their swelling ratio. Sample C-1 exhibited a relatively higher swelling ratio than other hydrogels. It showed the highest swelling ratio value of 784% at 1 h and 227%, 251%, 223%, and 222% at time intervals of 0.5, 2, 3, and 24 h, respectively. Sample C-2 showed a maximum absorption value of 593% at 2 h and 204%, 189% and 162%, and 178% at 0.5, 1, 3, and 24 h, respectively. Sample C-3 showed a maximum absorption value of 607% at 1 h, whereas 185%, 223%, 185%, and 185% swelling values were observed at 0.5, 2, 3, and 24 h, respectively. Sample C-4 showed the highest swelling ratio value of 457% at 1 h, whereas 199%, 205%, 172%, and 179% swelling values were observed at 0.5, 2, 3, and 24 h, respectively.

### 2.5. Thermogravimetric Analysis

**Figure 5:**
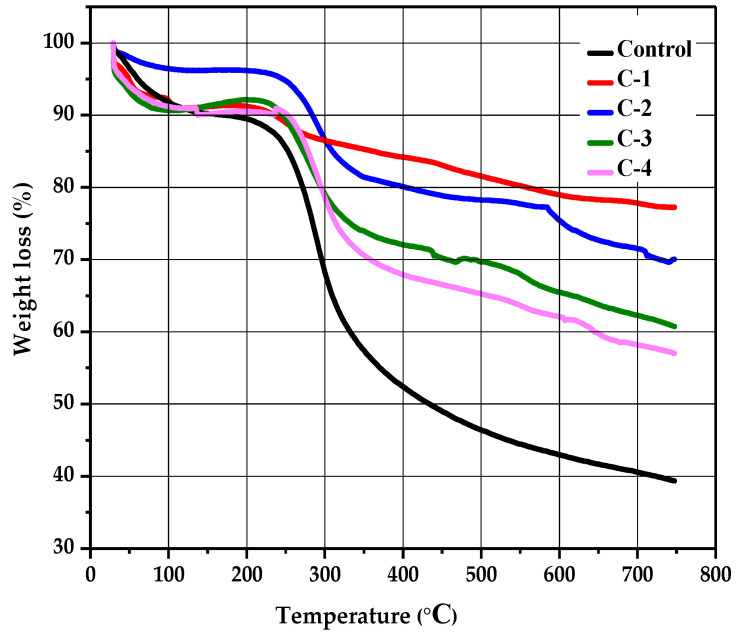
Thermogravimetric analysis of CS/*Cp*-LE-based hydrogels.

The stability and thermal degradation of the CS/*Cp*-LE hydrogels were studied using TGA analysis. Figure 5 shows the TGA curves of control for C-1, C-2, C-3, and C-4 hydrogels. A profile of TGA curves showed weight loss in three stages for all hydrogels. All hydrogel samples displayed the first degradation stage in a temperature range of 84 to 232 °C; this initial weight loss occurred due to the removal of water bound with CS and was 4% for control (CS) and 1% for all other CS/*Cp*-LE hydrogels. A second decomposition stage occurred from 224 to 339 °C (Figure 5), and the weight loss that occurred in the second stage was 28% for control, 3% for C-1, 10% for C-2, 15% for C-3, and 12% for C-4 hydrogel, and this stage corresponded to the oxidative and thermal decomposition of chitosan and removal of volatile products [35]. While comparing the second weight loss of the control hydrogel to C-1, C-2, C-3, and C-4 hydrogels, it was observed that the control hydrogel showed a 28% weight loss, which is higher than that observed for the other CS/*Cp*-LE hydrogels, possibly due to the crosslinking between CS and *Cp*-LE plant material. Of all hydrogels, C-1 showed a quite low weight loss of 3%.

There is also a third decomposition stage shown in the hydrogels, starting at 327 °C and ending at 621 °C, where weight loss occurred due to the char residue of 14% for the control group, while weight loss for the other CS/*Cp*-LE hydrogels was less than that in the control (Table 2). Char was formed during the second decomposition stage [36].

The C-1 hydrogel showed the highest thermal stability, and the control hydrogel showed the lowest thermal stability of all hydrogel samples. As the Cp-LE ratio of the hydrogel samples decreased, so did the thermal stability, as C-2 showed less thermal stability than C-1, C-3 showed less thermal stability than C-2, and so on. *Cp*-LE contents in the hydrogel impart stability possibly due to the strong interaction between the CS and Cp-LE blend that may protect them from decomposing during heat and reduce their mass loss during thermogravimetric analysis. For all hydrogels, Table 2 shows the starting region of thermal decomposition (T-onset) and end region (T-endset). W represents the percentage of weight loss.

### 2.6. Chorioallantoic Membrane (CAM) Assay

**Figure 6:**
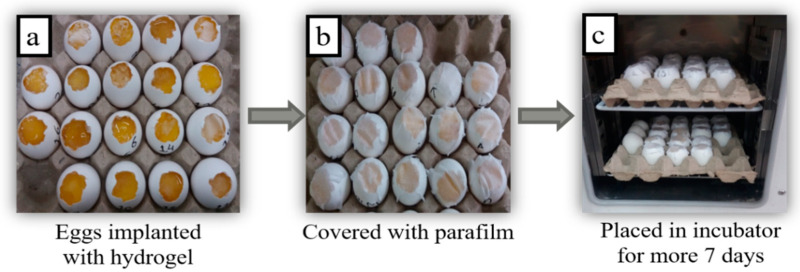
Stepwise handling of eggs for the CAM assay.

A CAM assay was executed to measure the healing capability of CS/*Cp*-LE hydrogels and the growth of new connective tissues and microscopic blood capillaries (angiogenesis). After the hydrogels were implanted on the cut surfaces of eggs, they were covered with parafilm and paper tape to prevent any fungal infection. All eggs were placed in the incubator at 37 °C. After the completion of 14 days, the eggs were removed from the incubator, their images were taken with a light microscope, and the response of the implanted hydrogels was examined on each egg (Figure 6).

**Figure 7:**
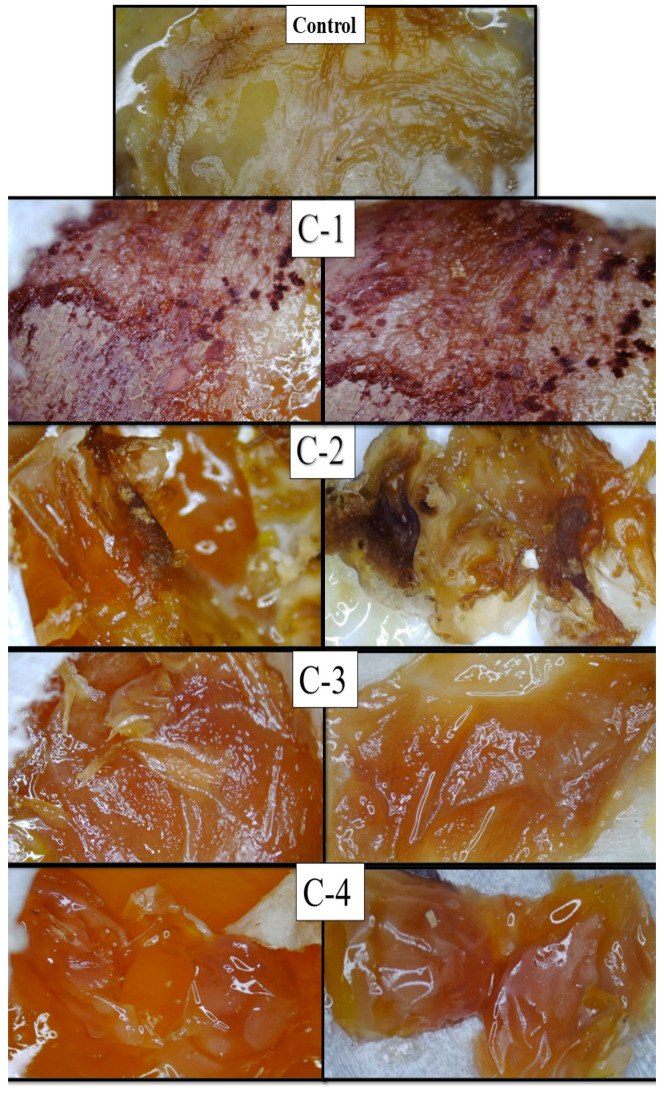
Microscopic images of fertilized eggs implanted with CS/*Cp*-LE hydrogels.

The microscopic images of eggs implanted with the control hydrogel (CS) exhibited much less healing, while eggs implanted with the hydrogel samples C-1, C-2, C-3, and C-4 displayed growth of regenerated cells and blood vessels compared to the control hydrogel. *Cp*-LE with CS exhibited significant healing properties. The C-1 hydrogel showed significantly enhanced formation of connective tissues and blood capillaries on the chorioallantoic membrane as compared to all CS/*Cp*-LE hydrogels after 14 days (Figure 7), while the C-2 hydrogel showed a thick formation of blood vessels. The C-3 and C-4 hydrogels showed a comparatively low formation of new cells and blood vessels as compared to the C-2 hydrogel, due to the low concentration of *Cp*-LE. As the Cp-LE ratio decreased in the hydrogels, the granulation tissue formation potential of hydrogel also decreased. Overall, it can be concluded that the CS/*Cp*-LE hydrogels showed good healing potential compared to the control sample.

Both chitosan and the *Calotropis procera* latex extract displayed a significantly high healing capability (Figure 7). In the previous literature, it has also been stated that *Calotropis procera* has wound healing properties. Rasik et al. [25] applied a latex solution of *Calotropis procera* on a guinea pig back. This increased the speed of tissue granulation, ensured increased blood vessel formation, and increased epithelization. Aderounmua et al. [37] applied *Calotropis procera* latex to rabbits for 21 days, which resulted in the formation of the granulation tissues and reduction of the wound site. The possible reason behind this mechanism might be the presence of phytochemicals such as tannins, flavonoids, and alkaloids in the latex of plants [38]. These are known to improve wound healing because of their antimicrobial and healing properties that increase epithelization rate and blood vessel formation and facilitate wound contraction [39]. Latex has especially been found to increase wound healing by encouraging a distinct growing formation of DNA, protein, and collagen [40]. However, in addition to the latex of *Calotropis* extract remarkably affecting healing at different stages of tissue repair, it seems that the concentration of latex extract plays a crucial role in healing capability.

## 3. Materials and Methods

### 3.1. Chemicals and Preparation Methods

Medical grade chitosan powder from chitin of crab shells was purchased from Daejung Chemical (Si-Heung, Korea). Degree of deacetylation (DD), viscosity (µ, 1% in 1% acetic acid, 20 °C), and viscosity average molecular weight (Mv) are equal to 82.6–87.5%, 351–750 mPas, and 310-375 kDa, respectively [41]. *Calotropis procera* plant extract was collected from plants located at the University of Agriculture, Faisalabad, Pakistan (31.4278° N, 73.0758° E). Absolute alcohol, sodium hydroxide (NaOH), sodium chloride (NaCl), and acetic acid (CH_3_COOH) were obtained from Sigma-Aldrich (Burlington, VT, USA).

#### 3.1.1. *Calotropis procera* Extract Preparation

Firstly, the latex of *Calotropis procera* (sodom apple) plant was collected by breaking the stem of the plants located behind the new science block of the University of Agriculture, Faisalabad, Pakistan (31.4278° N, 73.0758° E). After collection, the latex extract was slightly diluted and filtered using Whatman filter paper and left until complete filtration had taken place. After that, the filtrate was collected in a bottle and stored at 4 °C in a refrigerator [27].

#### 3.1.2. Preparation of Chitosan Solution

A 1% stock solution of chitosan was prepared by mixing 1 g of chitosan in 100 mL of distilled water. After that, two drops of 1% acetic acid were added [28]. Then the solution was placed on a magnetic stirrer for 12 h at room temperature for the complete mixing of chitosan, and two more drops of acetic acid were added after 6 h. After 12 h of stirring, a clear homogenized chitosan solution was obtained and stored in the refrigerator at 4 °C.

#### 3.1.3. Synthesis of CS/Cp-LE Hydrogel

Hydrogel was synthesized by mixing prepared CS solution and *Cp*-LE through freeze-thawing. The concentrations mentioned in Table 1 show the weight percent ratio of *Cp*-LE and CS solution.

These concentrations were poured into Petri dishes and stirred until the solution became clear and were then placed in the refrigerator at −20 °C for overnight. A three molar (3 M) solution of sodium hydroxide was made in distilled water. Frozen samples were then coagulated with sodium hydroxide solution and refrozen for 12 h at −20 °C. Then, the samples were washed two times with absolute alcohol and further washed two times with distilled water to neutralize the samples’ pH. In the end, neutral samples were thawed at 37 °C in the oven for 24 h.

### 3.2. Characterization of Hydrogels

#### 3.2.1. Fourier Transform Infrared Spectroscopy

A Fourier transform infrared spectroscopic analysis (FTIR, PerkinElmer Spectrum Two, OH, USA) was carried out for the study of the chemical structure and functional groups on the material’s surface. Analysis was conducted in a range of 4000–650 cm^−1^.

#### 3.2.2. Scanning Electron Microscope Imaging

Scanning electron microscope (JSM-5910, JEOL, Akishima, Tokyo, Japan) images of the synthesized hydrogels were taken to examine the surface morphology. The operating voltage was 10 kilovolts. The samples were coated with gold using a sputter coater.

#### 3.2.3. Swelling Analysis

A swelling analysis of synthesized gels was carried out in a NaCl saline solution at 37 °C. The three molar (3 M) solution of sodium chloride (NaCl) was prepared in distilled water. A considerable difference in weight between dry (W*_d_*) and wet (W) hydrogels was observed after specified intervals of 0.5, 1, 2, 3, and 24 h [29].

Equation (1) was used to measure the swelling percentage.

(1) $$\mathrm{Swelling}\;\left(\%\right)=\frac{W-W_{d}}{W_{d}}\times 100\;$$

#### 3.2.4. Thermogravimetric Analysis

A Netzsch TG 209F1 Libra thermogravimetric system was used to check the thermal stability of CS hydrogels incorporating *Cp*-LE. Samples were heated from 25 to 750 °C under a nitrogen flow of 20 mL/min at a heating rate of 10 °C per minute.

#### 3.2.5. CAM Assay

A CAM assay was carried out to determine the healing activity of the prepared hydrogels. At day zero, chick eggs were purchased from the bakery, carefully cleaned with absolute ethanol, and then placed in an incubator at 37 °C in a humid environment. After seven days, on day 8, sterilized hydrogels were implanted on the eggs, cutting off small pieces of each eggshell with a surgical blade. After that, the eggs were sealed with sterilized parafilm or covered by adhesive tape to stop any infection and returned to the incubator. Eggs were reopened on day 14, and their healing response images were captured with a light microscope to examine changes that occurred after implanting hydrogels [29].

## 4. Conclusions

The present research work explains the growth of new connective tissues and angiogenesis of chitosan-based hydrogels incorporating latex of *Calotropis procera*. Chitosan and latex extract were mixed through freeze-thawing, which is the best physical approach to synthesize hydrogel because no heating or reagents that may cause toxicity are required. SEM results demonstrate that higher ratios of *Cp*-LE create pores in hydrogels, and their porosity decreases in descending order of *Cp*-LE concentration. The synthesized CS/*Cp*-LE scaffolds displayed excellent swelling capacity over the control hydrogel, which might be due to the porous structure of hydrogel. The C-1 hydrogel presented the highest thermal stability of all hydrogels. It was noticed that the C-1 hydrogel showed the highest formation of granulation tissues and blood vessels compared with the control and other hydrogels. Overall, plant extract in combination with chitosan in the form of hydrogel exhibits significant healing properties that can be further utilized in wound healing applications.

## Figures and Tables

**Table 1:** Sample concentration of chitosan (CS) and *Calotropis procera* latex extract.

Samples	CS (Wt %)	*Cp*-LE (Wt %)
Control	100	nil
C-1	60	40
C-2	70	30
C-3	80	20
C-4	90	10

**Table 2:** TGA analysis of control, C-1, C-2, C-3, and C-4 hydrogels.

Sample	Stages	T-_onset_ (°C)	T-_endset_ (°C)	W (%)
Control	I	84.7	214.8	4
	II	249.8	339.8	28
	III	353.6	621.4	14
C-1	I	99.7	202.3	1
	II	224.8	259.8	3
	III	327.3	587.1	6
C-2	I	94.7	224.8	1
	II	259.8	319.2	10
	III	349.8	584.6	4
C-3	I	79.7	214.8	1
	II	244.9	324.8	15
	III	358.6	598.5	9
C-4	I	92.4	232.3	1
	II	257.3	327.3	12
	III	353.6	606.2	6

## Data Availability

The data presented in this study are available on request from the corresponding author.

## Sample Availability

Samples of the compounds are available from the author Maria Lodhi.
